# Hydroxychloroquine in the treatment of adult patients with Covid-19 infection in a primary care setting (LIBERTY): A structured summary of a study protocol for a randomised controlled trial

**DOI:** 10.1186/s13063-020-04989-6

**Published:** 2021-01-11

**Authors:** Petri J. Vainio, Pauliina Hietasalo, Anna-Liisa Koivisto, Susanna Kääriäinen, Jari Turunen, Mika Virtala, Jouni Vuorinen, Mika Scheinin

**Affiliations:** 1 Institute of Biomedicine, University of Turku, Turku, Finland; 2 Unit of Clinical Pharmacology, Turku University Hospital, Turku, Finland; 3 Porin perusturva, Pori, Finland; 4 Clinical Research Services Turku – CRST Oy, Turku, Finland; 5 Oy 4Pharma Ltd., Turku, Finland

**Keywords:** COVID-19, Randomised controlled trial, protocol, hydroxychloroquine, primary care, open care, public health care, hospitalization

## Abstract

**Objectives:**

The primary objective of this study is to evaluate the therapeutic potential of hydroxychloroquine (HCQ) in the treatment of adult patients with PCR-confirmed Covid-19 infection in a primary open-care setting, as compared to placebo. The study hypothesis is that treatment with HCQ will reduce the risk of hospitalization because of Covid-19 infection, and the sample size estimate of the study is based on the need to test this hypothesis.

The secondary objectives of the study are:

- to evaluate the safety and tolerability of HCQ in the treatment of adult patients with PCR-confirmed Covid-19 infection in a primary open-care setting, as compared to placebo;
- to collect experience of the use of HCQ in the treatment of Covid-19 infection in outpatients, in order to be able to identify patient characteristics that predict specific treatment responses (favourable or unfavourable); this objective will also be addressed by post-hoc subgroup analysis of the study results and by meta-analysis of pooled patient data from other clinical trials of HCQ in outpatients; and
- to evaluate the impact of Covid-19 infection and its treatment on the mental health and well-being of the study participants.

In addition, if the data allow, the study has the following exploratory objectives:

- to evaluate the extent and duration of SARS-CoV-2 viral shedding by PCR testing of nasopharyngeal swab samples in study subjects treated with HCQ, as compared to placebo;
- to evaluate the extent and time course of SARS-CoV-2 virus-specific antibody responses in serum of study subjects treated with HCQ, as compared to placebo;
- to evaluate other possible biomarker changes in blood in study subjects treated with HCQ, as compared to placebo;
- to explore the possible effects of genetic variation in drug metabolizing enzymes on HCQ-related outcomes in the study population;
- to explore the associations of HCQ-related outcome variables with other patient characteristics, e.g. HLA haplotypes, HCQ concentrations, demographic variables, disease history and concomitant medications.

**Trial design:**

This is a phase 2, placebo-controlled, double-blind, randomized, parallel-group treatment trial comparing HCQ with placebo in outpatients with Covid-19 infection. Participants will be randomized in a 1:1 ratio to the two treatment arms.

**Participants:**

Main inclusion criteria:

1. Males and females >40 years of age, or 18-40 years of age with one or both of the following: i. diabetes mellitus (type 1 or type 2); ii. BMI > 35 kg/m^2^;
2. Valid independent informed consent obtained;
3. Symptoms typical of Covid-19 infection, according to criteria specified in the study protocol. The onset of symptoms must be within 5 days of enrolment;
4. Positive SARS-CoV-2 PCR test result of a nasopharyngeal swab sample.

Main exclusion criteria:

1. Suspected severe or moderately severe pneumonia, presenting with any of the following: respiratory rate > 26 breaths/min; significant respiratory distress; or SpO_2_ ≤94% on room air;

2. Requiring treatment in the hospital, according to the treating physician’s judgement;

3. Any contraindication to treatment with HCQ;

4. Pregnancy or lactation.

The trial will be conducted at seven study sites in a primary public health care setting in the region of Satakunta, Finland.

**Intervention and comparator:**

Participants will be randomized to receive either HCQ capsules at 300 mg twice a day for one day and then 200 mg twice a day for 6 days, or placebo capsules for 7 days.

**Main outcomes:**

The primary endpoint of the study is the number of hospitalizations due to Covid-19 infection within four weeks of entry into the study.

The secondary endpoints of the study include the following:

- duration and severity of Covid-19-related symptoms, as reported by daily self-assessments;
- number of Intensive Care Unit treatment episodes due to Covid-19 infection within four weeks of entry into the study;
- number of deaths due to Covid-19 infection within four weeks of entry into the study;
- number of treatment-related adverse events (AEs) and serious AEs (SAEs);
- all-cause hospitalizations and mortality within six months of entry into the study; and
- self-assessed symptoms of anxiety, as assessed with repeated administration of the Generalized Anxiety Disorder 7-item scale (GAD-7).

The exploratory endpoints of the study include the following:

- extent and duration of SARS-CoV-2 viral shedding and virus-specific antibody responses in serum; and
- possible other blood biomarker changes.

**Randomisation:**

Eligible study participants are randomly allocated into two treatment arms (1:1 ratio). The randomization list has been generated using Viedoc™ (Viedoc Technologies AB, Uppsala, Sweden) that is used as an electronic data capture system for this study.

**Blinding (masking):**

The participants and all study personnel remain blinded to the treatment allocation by having both IMPs packed in identical containers. Masking of the treatments was performed by re-formulation of the IMPs so that the HCQ capsules and the placebo capsules have identical appearance.

**Numbers to be randomised (sample size):**

600 participants are to be randomised with 300 in each arm.

**Trial Status:**

Protocol version 2, dated 14 July 2020; recruitment is expected to start in December, 2020, and to be completed in June, 2021.

**Trial registration:**

EudraCT 2020-002038-33, registered 26 June 2020

**Full protocol:**

The full protocol is attached as an additional file, accessible from the Trials website (Additional file 1). The protocol has been redacted to conform with privacy regulations by deleting the names and contact information of individuals mentioned in the protocol but not listed as authors in this communication. In the interest of expediting dissemination of this material, the familiar formatting has been eliminated; this Letter serves as a summary of the key elements of the full protocol.

**Supplementary Information:**

The online version contains supplementary material available at 10.1186/s13063-020-04989-6.

## Supplementary Information

**Additional file 1.** Full Study Protocol.

## Data Availability

The study data will be available from the authors on reasonable request for meta-analysis and other purposes covered by the participants´ consent (contact information for requests: mschein@utu.fi).

