# Supplementary material for: Hydroxychloroquine in the treatment of adult patients with Covid-19 infection in a primary care setting (LIBERTY): A structured summary of a study protocol for a randomised controlled trial
Source: Trials. 2021 Jan 11;22:44. doi: 10.1186/s13063-020-04989-6 (PMC7797713; doi:10.1186/s13063-020-04989-6)
Supplement: Supplementary file 1 — Additional file 1. Full Study Protocol. [file 13063_2020_4989_MOESM1_ESM.pdf]

CLINICAL STUDY PROTOCOL

Controlled clinical trial of hydroxychloroquine in the treatment of adult patients with Covid-19 infection in a primary care setting

Study Code:	LIBERTY
EudraCT No:	2020-002038-33
Phase:	2
Sponsor:	City of Pori
Principal Investigator:	Adjunct Professor Petri Vainio, MD, PhD
Protocol Version:	Version 2
Protocol Date:	14Jul2020

The study will be conducted in accordance with GCP and applicable legislation and regulatory guidance.

SIGNATURES

Principal Investigator:	___________________________<br>Petri Vainio, MD, PhD	Date: _____/_____/________
Biostatistician:	__________________________<br>Jouni Vuorinen, PhD	Date: _____/_____/________
Representative of the Sponsor:	___________________________<br>Aino-Maija Luukkonen, Mayor	Date: _____/_____/________

CONTACT INFORMATION

Principal (Coordinating) Investigator, Person Responsible for the Study

Adjunct Professor Petri Vainio, MD, PhD
University of Turku, Institute of Biomedicine
Kiinamyllynkatu 10, FI-20520 Turku, Finland
Phone: +358 29 450 4657
Email: petri.vainio@utu.fi

Data Management and Biostatistics

Jari Turunen, MSc (Data Manager)
Jouni Vuorinen, PhD (Responsible Biostatistician)
Oy 4Pharma Ltd.
Tykistökatu 4 D
FI-20520 Turku, Finland
Phone: +358 2 283 5700

Safety Laboratory

SataDiag, Pori, Finland
@satadiag.fi, Sairaalantie 3, FI-28500 Pori,
Phone +358 2 627

Other laboratories

Diagnostic PCR testing : SataDiag, Pori, Finland

Antibody testing from serum: TYKSLAB and University of Turku, Institute of Biomedicine, Turku, Finland

HCQ concentration analysis: University of Turku, Institute of Biomedicine, Bioanalytical Laboratory (Director: Petri Vainio, MD, PhD, e-mail petri.vainio@utu.fi)

Genotyping for enzymes involved in drug metabolism: Abomics Oy, Tykistökatu 4 B, FI-20520 Turku, Finland

Genotyping for HLA haplotypes : TYKSLAB, Turku, Finland

Pharmacy Services

Satadiag, Hospital Pharmacy of Pori Central Hospital (
@satadiag.fi, Sairaalantie 3, FI-28500 Pori, Finland
Phone +358 627

Study Sites

- Euran sote-keskus (Terveystie 3, FI-27510 Eura, Finland), site principal investigator , MD
- Huittisten terveyskeskus (Risto Rytin katu 53, FI-32700 Huittinen, Finland), site principal investigator , MD
- KSTHKY (Keski-Satakunnan terveydenhuollon kuntayhtymä; Koulukatu 2, FI-29200 Harjavalta, Finland), site principal investigator MD
- Porin perusturva (Maantiekatu 31, FI-28100 Pori, Finland), site principal investigator MD,
- PoSa (Pohjois-Satakunnan peruspalvelukuntayhtymä; Tapalankatu 20, FI-38700 Kankaanpää, Finland), site principal investigator MD
- Rauman terveyspalvelut (Steniuksenkatu 2, FI-26100 Rauma, Finland), site principal investigator MD

- Säkylän terveyskeskus (Välskärintie 5, FI-27800 Säkylä, Finland), site principal investigator , MD

SAE Reporting

Investigators and study sites will be responsible for SAE reporting according to applicable regulatory guidance.

Sponsor's Medical Monitor/Medical Lead: , MD, Porin perusturva
Maantienkatu 31, FI-28120 Pori, Finland
e-mail @fimnet.fi,
Phone +358 40

Monitoring

Study monitoring will be carried out by Turku Clinical Research Centre (TurkuCRC, see www.turkucrc.fi)

Steering Committee

Chair : Aino-Maija Luukkonen, PhLic, MPH, Mayor, City of Pori (Sponsor's representative)

Secretary : Porin perusturvayhtymä

Members :

Chief Physician,

Professor,

Professor,

Mika Scheinin, MD, PhD, Professor emeritus, University of Turku, Chief Scientific Officer, CRST Oy (clinical pharmacology, clinical trials, research ethics)

Chief Physician,

Study Coordinators

Porin perusturvayhtymä (logistics, training)

Porin perusturvayhtymä (study management, safety surveillance, Medical Monitor/Medical Lead of the Sponsor)

TABLE OF CONTENTS

SYNOPSIS 7
ABBREVIATIONS AND DEFINITION OF TERMS 14

1 INTRODUCTION 16
1.1 Background 16
1.2 Rationale of the study 18
1.3 Risk benefit assessment 18
2 STUDY OBJECTIVES AND ENDPOINTS 18
2.1 Primary objective 18
2.2 Secondary objectives 19
2.3 Exploratory objectives 19
2.4 Primary endpoint 19
2.5 Secondary endpoints 20
2.6 Exploratory endpoints 20
3 STUDY DESIGN 20
3.1 Type and design of the study 20
3.2 Randomization and blinding 20
3.3 General study outline 21
4 STUDY PATIENTS/SUBJECTS 23
4.1 Source population 23
4.2 Number of study patients/subjects 23
4.3 Inclusion criteria 23
4.4 Exclusion criteria 23
4.5 Recruitment 24
4.6 Instructions concerning lifestyle and concomitant treatments 25
4.7 Withdrawal of subjects 25
5 TREATMENTS 25
5.1 Investigational treatment 25
5.2 Reference treatment 26
5.3 Handling of the study products 26
5.4 Prior and concomitant treatments 26
5.5 Procedures for monitoring of subject compliance 26
6 VISIT SCHEDULE 26
6.1 Screening 26
6.2 Treatment period 27
6.3 Follow-up period 28
7 ASSESSMENTS 28
7.1 Screening data 28
7.1.1 Demographic and other baseline information 28
7.1.2 Screening laboratory safety determinations 29
7.1.3 Covid-19 related laboratory assessments 29
7.2 Assessment of efficacy 29
7.2.1 Evaluation of patient records 29
7.2.2 Patient-reported health outcomes 30
7.2.3 SARS-CoV-2 PCR analysis 30
7.2.4 Analysis of antibodies and biomarkers 30
7.3 Assessment of safety 30
7.3.1 Clinical safety assessments 30
7.3.2 Laboratory safety assessments 30
7.3.3 Adverse events 31
7.4 Assessments of pharmacokinetics, pharmacogenetics and immunogenetics 33

8 STATISTICS AND DATA MANAGEMENT 33
8.1 Estimation of sample size 33
8.2 Statistical methods 33
8.2.1 Statistical Analysis Plan 34
8.2.2 Statistical hypotheses 34
8.2.3 Data sets to be analyzed 34
8.2.4 General statistical considerations 34
8.2.5 Demographic and baseline characteristics 34
8.2.6 Analysis of efficacy variables 34
8.2.7 Analysis of safety variables 35
8.2.8 Analysis of pharmacokinetic, pharmacogenetic and immunogenetic variables 35
8.3 Data management 35
8.3.1 Database design 35
8.3.2 Data entry 36
8.3.3 The query process 36
8.3.4 Medical encoding 36
8.3.5 Database lock 36
8.4 Software 36
9 QUALITY CONTROL AND QUALITY ASSURANCE 36
10 ETHICAL CONSIDERATIONS 37
11 DATA HANDLING AND RECORD KEEPING 38
11.1 Case report forms 38
11.2 Source data 38
11.3 Deviations 38
11.4 Amendments 39
12 STUDY SCHEDULE 39
13 CRITERIA FOR PREMATURE STUDY TERMINATION 39
14 FINANCING AND INSURANCE 40
15 STUDY REPORT AND PUBLISHING 40
16 ARCHIVING 40

REFERENCES 40
APPENDICES 41

SYNOPSIS

**Name of the Sponsor**<br>City of Pori		
**Name of the test product**<br>Oxiklorin 100 mg tablets, manufactured by Orion Pharma and encapsulated by the SataDiag hospital pharmacy		
**Name of the active ingredient**<br>Hydroxychloroquine sulphate		

Study code

EudraCT 2020-002038-33; LIBERTY

Study title

Controlled clinical trial of hydroxychloroquine in the treatment of adult patients with Covid-19 infection in a primary care setting

Investigators and study centres

Coordinating Investigator: Petri Vainio, MD, PhD, Adjunct Professor of Pharmacology and Drug Development, University of Turku, Institute of Biomedicine

Study sites and their principal investigators:

- Euran sote-keskus (Terveystie 3, FI-27510 Eura, Finland), site principal investigator , MD
- Huittisten terveyskeskus (Risto Rytin katu 53, FI-32700 Huittinen, Finland), site principal investigator , MD
- KSTHKY (Keski-Satakunnan terveydenhuollon kuntayhtymä; Koulukatu 2, FI-29200 Harjavalta, Finland), site principal investigator MD,
- Porin perusturva (Maantiekatu 31, FI-28100 Pori, Finland), site principal investigator MD
- PoSa (Pohjois-Satakunnan peruspalvelukuntayhtymä; Tapalankatu 20, FI-38700 Kankaanpää, Finland), site principal investigator MD
- Rauman terveyspalvelut (Steniuksenkatu 2, FI-26100 Rauma, Finland), site principal investigator MD
- Säkylän terveyskeskus (Välskärintie 5, FI-27800 Säkylä, Finland), site principal investigator MD

Development phase

2

Objectives and endpoints

This placebo-controlled, randomized, parallel-group clinical trial aims to investigate the safety, tolerability and therapeutic potential of hydroxychloroquine sulphate (HCQ) in the treatment of adult patients with PCR-confirmed Covid-19 infection in a primary care setting. The study is first started in the region of Satakunta in Finland (pop. 217.000), but it may later be expanded to other regions of Finland. HCQ has been in widespread compassionate use in the treatment of patients with Covid-19 infection, but evidence of its usefulness for this purpose is still lacking. Randomized controlled trials (RCT) have been initiated to investigate its efficacy and safety in hospitalized, moderately or severely ill patients. The present RCT is targeting an earlier and milder stage of the disease than the hospital-based trials, i.e. adult patients seeking medical care for Covid-19 infection in a primary health care setting. A placebo-controlled randomized trial of HCQ in this patient population is considered to be warranted and ethically justified, as no established specific treatments are available for this novel and potentially lethal viral disease, as HCQ may

have potential to modify the course of the Covid-19 disease in its early phase, and as the Finnish public health care system and Finland’s population-based registries provide a unique platform to test this approach to address a very urgent unmet medical need.

The primary objective of the study is:
- to evaluate the therapeutic potential of HCQ, dosed at 300 mg b.i.d. on the first day and then 200 mg b.i.d. for six days (total dose, 3000 mg), in the treatment of adult patients with PCR-confirmed Covid-19 infection in a primary open-care setting, as compared to placebo.

The secondary objectives of the study are:
- to evaluate the safety and tolerability of HCQ, dosed at 300 mg b.i.d. on the first day and then 200 mg b.i.d. for six days (total dose, 3000 mg), in the treatment of adult patients with PCR-confirmed Covid-19 infection in a primary open-care setting, as compared to placebo;
- to collect experience of the use of HCQ in the treatment of Covid-19 infection in outpatients, in order to be able to identify patient characteristics that predict specific treatment responses (favourable or unfavourable); this objective will also be addressed by post-hoc subgroup analysis of the study results and by meta-analysis of pooled patient data from other clinical trials of HCQ in outpatients. The subjects’ informed consent covers such further use of the study data; primarily, meta-analysis has been planned with two investigator-initiated open-care trials of HCQ carried out in Germany (see COMIHY, www.ClinicalTrials.gov NCT04340544, and COVID65plus, NCT04351516); and
- to evaluate the impact of Covid-19 infection and its treatment on the mental health and well-being of the study participants.

In addition, if the data allow, the study has the following exploratory objectives:
- to evaluate the extent and duration of SARS-CoV-2 viral shedding, as evaluated with PCR testing of nasopharyngeal swab samples in study subjects treated with HCQ, as compared to placebo;
- to evaluate the extent and time course of SARS-CoV-2 virus-specific antibody responses in serum of study subjects treated with HCQ, as compared to placebo;
- to evaluate other possible biomarker changes in blood in study subjects treated with HCQ, as compared to placebo;
- to explore the possible effects of genetic variation in drug metabolizing enzymes on HCQ-related outcomes in the study population;
- to explore the associations of HCQ-related outcome variables with other patient characteristics, e.g. HCQ concentrations in blood, demographic variables, genetic variation in the immune system, disease history and concomitant medications.

The primary endpoint of the study is:
- number of hospitalizations due to Covid-19 infection within four weeks of entry into the study; the study hypothesis is that treatment with HCQ will reduce the risk of hospitalization because of Covid-19 infection, and the sample size estimate of the study is based on the need to test this hypothesis.

The secondary endpoints of the study are:
- duration and severity of Covid-19-related symptoms, as reported by the participants in the form of daily self-assessments;
- number of Intensive Care Unit treatment episodes due to Covid-19 infection within four weeks of entry into the study;
- number of deaths due to Covid-19 infection within four weeks of entry into the study;

- number of hospitalizations due to Covid-19 infection within three months of entry into the study;
- number of Intensive Care Unit treatment episodes due to Covid-19 infection within three months of entry into the study;
- number of deaths due to Covid-19 infection within three months of entry into the study;
- number of treatment-related adverse events (AEs) and serious AEs (SAEs);
- number of participants with treatment-related AEs and SAEs;
- all-cause mortality within six months of entry into the study (including post-study follow-up of consenting participants);
- all-cause hospitalizations within six months of entry into the study (including post-study follow-up of consenting participants); and
- self-assessed symptoms of anxiety of the study participants, as assessed with repeated administration of the Generalized Anxiety Disorder 7-item Scale (GAD-7).

The exploratory endpoints of the study are:

- extent and duration of SARS-CoV-2 viral shedding, as evaluated with PCR testing of nasopharyngeal swab samples;
- extent and time course of SARS-CoV-2 virus-specific antibody responses in serum; and
- possible other biomarker changes in blood in study subjects.

For the purposes of the present study, “patients with PCR-confirmed Covid-19 infection” denotes subjects clinically diagnosed with probable Covid-19 infection AND at least one positive SARS-CoV-2 virus test result in PCR analysis of a nasopharyngeal swab sample, taken within five days of study entry. Treatment with study drugs may only be started once a positive SARS-CoV-2 test result in PCR analysis of a nasopharyngeal swab sample has been obtained. The study results will be analysed for three separate datasets: an intention-to-treat (ITT) dataset including all enrolled subjects; a safety dataset including all subjects receiving at least one dose of study medication; and a per-protocol (PP) dataset including all subjects with SARS-CoV-2 PCR-confirmed Covid-19 infection who received at least four days of treatment with study medication and for whom evaluable outcome data are available. Further details are provided in the study protocol under the headings “Subject eligibility criteria” and “Statistical analysis plan”.

Methodology

This is a phase 2, placebo-controlled, double-blind, randomized, parallel-group treatment trial of HCQ in patients with PCR-confirmed Covid-19 infection, performed at several study sites located in Finland, in a primary public health care setting. The participating patients will be randomized into one of two treatment groups: HCQ capsules for seven days (total dose: 3000 mg), or placebo capsules for seven days. The participants and study personnel will remain blinded to the treatment allocation by masking of the capsules and by having both investigational medicinal products (IMPs) packed in identical containers. The IMPs will be dispensed to the participants immediately upon enrolment and randomization, with instructions on their use, with instructions to contact the study site in case of any suspected AEs or worsening of symptoms, and with instructions regarding subsequent study-related procedures. As all screening test results will not be available at the screening visit, the participants will be contacted by telephone on the next day (or as soon as the screening test results are available) and told whether or not they are eligible for enrolment. Health-related instructions will be given, and eligible subjects will be told that they should start to use their study medication immediately upon its delivery. Home delivery of the medication will be arranged by the study sites.

Study participation consists of two phases: a treatment period and a follow-up period. Possibly eligible persons who contact the study sites to seek medical advice and treatment for possible Covid-19 infection will be informed about the study. A screening visit will be scheduled for possibly eligible subject candidates. At this visit, informed consent will be recorded in writing, and subject candidates will be evaluated for eligibility. Subjects may be enrolled and randomized, and treatment with study drugs may be started once the results of the screening assessments are available. A positive test result in SARS-CoV-2 PCR analysis of a nasopharyngeal swab sample taken and analysed outside of this study may be used for evaluation of virus positivity, if taken within five days of enrolment. Enrolment of subjects without previous PCR-confirmed SARS-CoV-2 virus shedding must have to await a positive SARS-CoV-2 PCR test result of a nasopharyngeal swab sample collected at the screening visit. Enrolment and randomization will be performed and IMP will be dispensed and delivered to study subjects only after the investigator has confirmed the subject’s eligibility.

During the treatment period, all participating patients will receive standard care for their Covid-19 infection, according to local treatment practice, which includes daily telephone contacts for evaluation of symptoms. In addition, they will receive study medication to take at home, 300 mg of HCQ to be taken twice daily on the first day, and then 200 mg b.i.d. for six days (Days 2-7), or corresponding placebo capsules. On Day 8, they will be contacted by telephone for an interview on symptoms and possible AEs. Approximately 2 weeks and 4 weeks after start of drug intake, the subjects will visit their study site for assessments. The participants will be instructed to contact the study site at any time during the treatment period if their condition worsens, if they experience a serious or significant AE or if they are hospitalized for any reason. The subjects will be provided with a participant card containing study-related instructions and emergency contact information for health care personnel that might be involved in the subjects’ subsequent medical care.

The follow-up period will start at the 4-week visit and will last until 3 months after start of IMP intake. Study participation will be concluded with a follow-up telephone contact approximately 3 months after the start of IMP intake. The following information will be collected from the following sources during the follow-up period: self-reported health outcomes, information on services received in public health care in Finland (Care Register for Health Care, see https://thl.fi/en/web/thlfi-en/statistics/information-on-statistics/register-descriptions/care-register-for-health-care#name), and information contained in death certificates archived by Statistics Finland (see https://www.stat.fi/tup/kuolintodistusarkisto/index_en.html). With the participants’ consent, additional post-study follow-up information will be collected from the Care Register for Health Care and from death certificates until 6 months after start of IMP intake, in order to allow evaluation of possible late-appearing adverse outcomes.

Number of Subjects

Approximately 600 subjects are planned to be included in the study by the summer of the year 2021, but actual enrolment numbers will depend on the development of the Covid-19 epidemic in Satakunta and in other participating regions of Finland. Enrolment will be discontinued prematurely if new information becomes available that mandates study discontinuation, e.g. for futility or for proof of efficacy of HCQ or another type of treatment of Covid-19 infection in the patient population targeted by this study. Also, enrolment may have to be terminated at any participating study site if sufficient resources can no longer be allocated to ensure adequate study conduct and participant safety. A Steering Committee (SC) will provide oversight of the study. One of the tasks of the SC will be to provide recommendations to the Sponsor and the Coordinating Investigator on whether enrolment of participants into the study should be continued or terminated, or whether modifications should be introduced into the study protocol in the form of a substantial

amendment. The SC and the data management (DM) provider of the trial will perform unblinded follow-up of all hospitalizations and deaths occurring in the study population in order to be able to call for termination or modification of the trial. No other interim analysis of the accumulating study results has been planned.

Main eligibility criteria

Diagnostic and main eligibility criteria of study participants are provided below.

Main inclusion criteria:

1. Subjects over 40 years of age, or 18-40 years of age with one or both of the following:
    a. diabetes mellitus (type 1 or type 2)
    b. BMI > 35 kg/m^2.
2. Subjects capable of providing independent informed consent and signing the informed consent form (the subjects' capacity to consent should be determined in accordance with applicable professional standards and will be based on the investigator's judgment).
3. Subjects with symptoms typical of Covid-19 infection, according to criteria specified in the study protocol, with or without known or suspected exposure to the SARS-CoV-2 virus. The onset of symptoms must be within 5 days of enrolment. Study medication may only be started once a positive SARS-CoV-2 PCR test results of a nasopharyngeal swab sample is available; if the first PCR test result is negative, a second sample may be collected and analysed.
4. Female subjects of child-bearing potential must agree to employ a reliable method of contraception until three months after start of IMP intake.
5. Subjects must agree not to share medication with others.

Main exclusion criteria:

1. Subjects with suspected severe or moderately severe pneumonia, presenting with any of the following: respiratory rate > 26 breaths/min; significant respiratory distress; or SpO_2 ≤ 94% on room air.
2. Subjects requiring treatment in the hospital, according to the treating physician's judgement.
3. Subjects having any contraindication to treatment with HCQ, such as a long QTc interval (>450 ms in men, >470 ms in women), porphyria, epilepsy, severe renal insufficiency;
4. Screening 12-lead ECG results showing evidence of clinically significant disturbances of cardiac rhythm or impulse conduction. Atrial fibrillation, paroxysmal supraventricular tachycardia or supraventricular extrasystoles are not exclusionary;
5. Cardiac insufficiency (NYHA Class ≥ 3);
6. History of stroke, within 6 months of screening;
7. Subjects with a history of generalized seizures within one year of screening;
8. Subjects with a history of drowning accident;
9. Any psychiatric diagnosis or symptoms (e.g., hallucinations, major depression, delusions, schizophrenia, bipolar disorder) that, in the opinion of the investigator, could interfere with study procedures or assessments or subject safety; prior history of suicidal behaviour or suicide attempt within five years of screening will also exclude the subject;
10. Disorder related to alcohol or drug abuse, as defined in DSM-5-TR, within one year prior to screening;
11. Evidence of current or history of any significant autoimmune disease that, in the opinion of the investigator, could interfere with evaluation of the study results or constitute a health hazard for the subject;

12. Evidence of an immune system that is compromised; including, but not limited to, a diagnosis of HIV; or the subject has been splenectomised or has received an organ transplant (corneal transplants excluded), or is receiving chronic systemic immunosuppressive medication;
13. Evidence of current unstable pulmonary, gastrointestinal, renal, hepatic, endocrine, haematological or cardiovascular system disease or metabolic disturbance;
14. Diagnosis of cancer (haematological or solid tumour) for which the subject is currently being treated, or for which there is evidence of active disease. Subjects with local prostate cancer or local dermatological tumours, such as basal or squamous cell carcinoma, may be included;
15. Any clinically significant abnormalities in laboratory tests, vital signs, ECG or physical examination findings at screening that in the opinion of the investigator may interfere with study procedures or safety. These may include, but are not limited to, the following:
    a. estimated glomerular filtration ratio (eGFR) < 30 ml/min/1.73 m^2, based on the CKD-EPI equation;
    b. a plasma total bilirubin value >2 times the upper limit of the reference range;
    c. plasma alanine aminotransferase (ALT) or aspartate aminotransferase (AST) value >3 times the upper limit of the reference range;
16. Current or anticipated use or recent prior use (pre-study time limits specified in the main protocol text) of disallowed concomitant treatment; use of concomitant medications that prolong the QTc interval is not allowed;
17. Having received in another clinical trial any other investigational medication (unless it can be documented that the subject received only placebo) within 1 month or 5 half-lives (whichever is longer) before screening;
18. Disease or medication that, in the opinion of the investigator, could seriously impact the assessments of safety, tolerability or efficacy;
19. Pregnancy or breast-feeding.

In addition to these eligibility criteria, subject safety will be ensured with close follow-up of symptoms and by following specific dosing discontinuation criteria, listed in the main protocol text.

Investigated treatment, dose and mode of administration

HCQ capsules will be dispensed to the study participants to allow dosing according to the following regimen: 300 mg b.i.d. on the first day and then 200 mg b.i.d. for six days (total dose, 3000 mg), to be taken with meals. IMPs will be re-formulated, packed and labeled for dispensing by the hospital pharmacy of Satakunta Central Hospital (SataDiag).

Placebo, dose and mode of administration

Placebo capsules will be used as control treatment, and dispensed to the study subjects in identical containers as HCQ, to be taken twice daily with meals, for seven days. The placebo capsules will be formulated, packed and labeled for dispensing by the hospital pharmacy of Satakunta Central Hospital (SataDiag).

Duration of treatment

IMPs will be taken twice daily by mouth, with meals, for seven consecutive days. Because of the slow elimination of HCQ, the treatment period of this study is defined as starting from the first IMP intake and ending at the 28-day follow-up visit.

Assessments

The efficacy, safety, tolerability and other assessments included in this study are listed below. The time points of the assessment are listed in the study schedule and in the main text of the protocol.

Efficacy, safety and tolerability will be evaluated using the following assessments:
- patient-reported health outcomes;
- answers to the Generalized Anxiety Disorder 7-item Scale (GAD-7) questionnaire;
- solicited and non-solicited AEs;
- SARS-CoV-2 PCR analysis of nasopharyngeal swab samples collected at screening and at the 2- and 4-week visits;
- analysis of SARS-CoV-2 antibody titres in serum samples collected at the screening and the 2- and 4-week visits;
- analysis of other Covid-19-related biomarkers in serum or plasma samples collected at the screening and the 2- and 4-week visits;
- clinical laboratory tests from blood samples collected at the screening and the 2- and 4-week visits;
- vital signs (including body temperature, blood pressure, heart rate, SpO_2 and respiratory rate);
- ECG recordings;
- physical examination findings, including body weight and chest auscultation findings;
- evaluation of patient records, including information collected from health-related registers (the Care Register for Health Care and death certificates).

Treatment compliance will be evaluated by asking the participants to register any deviations from the planned treatment regimen in a participant diary to be collected at the 2-week follow-up visit and by performing concentration analysis of HCQ in blood samples collected at the 2- and 4-week follow-up visits.

Statistical methods

The following analysis sets will be used to analyse and present the study results:
- Intention-to-treat (ITT): all randomized subjects;
- Safety analysis set: all subjects receiving at least one dose of study medication;
- Per protocol set (PP): all randomized subjects with SARS-CoV-2 PCR-confirmed Covid-19 infection who received at least four days of treatment with study medication and for whom evaluable outcome data are available.

For the primary variable of the study, the proportion of hospitalizations will be estimated within each treatment group. The main analysis will be done using a logistic regression model. The methods of analysis of all efficacy variables will be provided in the SAP. At least, analysis of all primary and secondary efficacy variables will be performed for both the PP and ITT datasets.

<u>Analysis of safety assessments:</u>
All safety results will be evaluated for the safety analysis set. Descriptive statistics will be used to summarise AEs, clinical safety laboratory test results, vital signs and body weight, ECG findings, and physical examination results.
<u>Sample size considerations:</u>
The number of hospitalizations due to Covid-19 infection within four weeks of entry into the study is a main outcome measure of the current study. Assuming a hospitalization rate of 15 % in the study population, and a 50 % reduction in the risk for hospitalization in the group treated with HCQ, a treatment group size of 278 provides 80 % power to detect a statistically significant treatment effect at a two-sided $p < 0.05$. As the actual risk of hospitalization in the study population is unknown, and also because a treatment effect

size of less than 50 % is considered potentially clinically significant, both expansion of the trial beyond the initial catchment region of Satakunta and meta-analysis by combining the study results with those derived from other similar patient populations are planned.

ABBREVIATIONS AND DEFINITION OF TERMS

ADL	Activities of daily living
ADR	Adverse drug reaction
AE	Adverse event
ALT	Alanine aminotransferase
AST	Aspartate aminotransferase
AUC	Area under the concentration by time curve
b.i.d.	Twice a day
BMI	Body mass index
CA	Competent authority
C_{max}	Maximum observed concentration
Covid-19	Coronavirus disease 2019
CRF	Case report form
CRP	C-reactive protein
DM	Data management
DSM-5-TR	The Diagnostic and Statistical Manual of Mental Disorders, Fifth Edition, Text Revision
EC	Ethics committee
ECG	Electrocardiogram
eCRF	Electronic case report form
eGFR	Glomerular filtration ratio, estimated
GAD-7	Generalized Anxiety Disorder 7-item Scale
GCP	Good Clinical Practice
GMP	Good Manufacturing Practice
HCQ	Hydroxychloroquine sulphate
ICF	Informed consent form
IMP	Investigational medicinal product
ISF	Investigator’s study file
ITT	Intention-to-treat
NIH	The National Institutes of Health
NIAID	National Institute of Allergy and Infectious Disease
NYHA	New York Heart Association
PCR	Polymerase chain reaction
PG	Pharmacogenetic
P-IL6	Plasma interleukin 6
PP	Per-protocol
PT	Preferred term
PV	Pharmacovigilance
RCT	Randomized controlled trial
SAE	Serious adverse event
SARS-CoV-2	Severe acute respiratory syndrome coronavirus 2
SC	Steering Committee
SD	Standard deviation
SOC	System organ class
SpO_2	Oxygen saturation
SUSAR	Suspected unexpected serious adverse drug reaction

1 INTRODUCTION

1.1 Background

Covid-19

COVID-19, a disease caused by SARS-CoV-2 coronavirus, appeared first in China in December 2019 (Lu et al., 2020) and has since spread around the world. The clinical presentation of COVID-19 infection ranges from asymptomatic infection to severe pneumonia that can lead to respiratory failure or death (Deshmukh et al., 2020; Li et al., 2020). Current treatment guidelines for COVID-19 infection by the National Institutes of Health (NIH) focus on infection prevention and control measures and supportive care, such as oxygen supply and mechanical ventilation for severely or critically ill patients (NIH COVID-19 Treatment Guidelines, 2020). On 29Apr2020, the NIH's National Institute of Allergy and Infectious Disease (NIAID) announced that Gilead Sciences' Ebola drug candidate remdesivir outperformed placebo in a clinical COVID-19 trial sponsored by the agency. The study met its primary outcome of statistically significant improvement in time to recovery by day 29 in patients hospitalized for COVID-19 infection. Patients treated with remdesivir showed a median time to recovery of 11 days compared with 15 days for those who received placebo - a 31 % faster time to recovery. The findings also showed a mortality rate of 8.0 % for the patients treated with remdesivir, vs. 11.6 % for the placebo group, suggesting a survival benefit to the nucleotide prodrug (Beigel et al., 2020). On 01May2020, the U.S. Food and Drug Administration (FDA) issued an Emergency Use Authorization for remdesivir to treat adults and children with severe COVID-19. Remdesivir must be administered intravenously, which represents a limitation to its use. The NIH Panel now recommends using remdesivir for the treatment of COVID-19 in hospitalized patients with severe disease, defined as SpO2 ≤94 % on ambient air, requiring supplemental oxygen, mechanical ventilation, or extracorporeal membrane oxygenation.

Hydroxychloroquine sulphate

Hydroxychloroquine sulphate (HCQ), an old anti-inflammatory and antimalarial drug, has been in wide compassionate use in the treatment of patients with COVID-19 infection, and e.g. the FDA initially issued an Emergency Use Authorization that allowed the off-label use of HCQ and chloroquine in treating COVID-19 patients (Rome et al., 2020), but later revised this position and cautioned against their use for COVID-19 outside the setting of a hospital or clinical trial. Indeed, HCQ has shown effectiveness in inhibiting SARS-CoV-2 infection in vitro (Liu et al., 2020), but RCT-level clinical evidence of its usefulness for the treatment of COVID-19 infection is so far lacking.

In the first published open-label non-randomised clinical trial of HCQ (Gautret et al., 2020), the results were encouraging. HCQ was associated with viral load reduction in nasopharyngeal swab samples of COVID-19 patients, and the effect was reinforced by concomitantly taken azithromycin. In that study, 26 patients with confirmed COVID-19 infection were treated with HCQ 200 mg t.i.d. for ten days (total dose, 6000 mg), and azithromycin was added to the treatment based on the investigator's clinical judgement. Another 16 patients who fulfilled an exclusion criterion or refused the treatment, or were from another centre where HCQ was not used, were included as controls. Virological clearance at day 6 post-inclusion was the primary endpoint of the study. The proportion of virologically cured patients at

day 6 was 100 % in the HCQ-azithromycin group, 57 % in the HCQ-only group, and only 12.5 % in the control group.

Some small randomized placebo-controlled trials (RCTs) have been performed to investigate HCQ in the treatment of hospitalized COVID-19 patients, but their reports have so far not been peer-reviewed and their quality of evidence appears less than perfect. In a Chinese RCT of 30 only patients (Chen JLL et al., 2020), no clinical benefit was demonstrated with HCQ at 400 mg per day for 5 days. In another Chinese RCT with 62 patients (Chen Z et al., 2020), treatment with HCQ (400 mg per day for 5 days) shortened the time to clinical recovery and promoted the absorption of pneumonia, compared to placebo-treated patients.

Also warnings against the use of HCQ have been issued. In a retrospective study based on 386 patients' hospital records (Magagnoli et al., 2020), the use of HCQ with or without azithromycin was not associated with reduced risk of death or need for mechanical ventilation in patients hospitalized with laboratory-confirmed SARS-CoV-2 infection. Instead, increased overall mortality was observed in the patients treated with HCQ alone compared to the patients treated with standard care. In this study population, HCQ was more likely to be prescribed to severely ill patients than to patients with milder clinical presentation. In another retrospective cohort study of 1438 patients hospitalized in metropolitan New York, compared with treatment with neither drug, the adjusted hazard ratio for in-hospital mortality for treatment with HCQ alone was 1.08, for azithromycin alone was 0.56, and for combined HCQ and azithromycin was 1.35. None of these hazard ratios were statistically significant. Thus, treatment with HCQ was not associated with reduced mortality among patients hospitalized with COVID-19 (Rosenberg et al., 2020).

Further, in a retrospective registry analysis of 96 000 hospitalized patients, HCQ alone and in combination with a macrolide not only failed to show efficacy in COVID-19 disease but was associated with an increased risk of ventricular arrhythmia and increased mortality (Mehra et al., 2020). Also this study was retrospective and register-based, and there was a risk of selection bias in the treatment allocation and in the reporting of adverse effects. The same caveats apply to the retrospective patient and ECG data analysis of Mercuro et al. (2020), where treatment with HCQ (with or without concomitant macrolide) was associated with an increased risk of QT interval prolongation. Those patients receiving concomitant azithromycin had a greater median (interquartile range) change in QT interval (23 [10-40] ms) compared with those receiving HCQ alone (5.5 [−15.5 to 34.25] ms). Most of the 90 patients included in the analysis had at least one cardiovascular comorbidity and were taking two or more QTc-prolonging medications, but only one of the patients (receiving both HCQ and azithromycin) presented with torsades de pointes tachycardia. These retrospective studies only evaluated hospitalized patients and thus included severely ill people with advanced forms of the disease, and it remains possible that the benefit-risk profile of HCQ may be substantially different in an early phase of the infection, in a patient population with mild to moderate disease treated in an outpatient setting.

Thus, the results of previous studies investigating the usefulness of HCQ in treating COVID-19 have been conflicting and non-conclusive, and more information is needed. Several RCTs of HCQ are currently ongoing. All such trials should be registered in publicly available registries, mainly https://www.who.int/ictrp/en/, https://clinicaltrials.gov/ or http://www.chictr.org.cn/abouten.aspx (Chinese Clinical Trial Registry); these registries were reviewed when preparing the current LIBERTY study protocol. Most ongoing trials are investigating the use of HCQ in hospitalized

patients or as preventive treatment for healthy individuals with known or suspected exposure to the SARS-CoV-2 virus, and there is an obvious need for studies to be performed with outpatients diagnosed at an early stage of the COVID-19 disease, in primary health care settings.

1.2 Rationale of the study

There is a high medical need for treatments for Covid-19 infection, as no established specific treatments are yet available. HCQ is used for treatment of Covid-19 patients, but proof of its usefulness for this purpose is lacking. The use of a drug such as HCQ without adequate clinical evidence of its efficacy and safety is in conflict with the principles of evidence-based medicine, particularly as the drug has well-documented risks. Predisposing patients to the risks of HCQ treatment is not justifiable without conclusive proof of clinical benefit (Mehra et al., 2020; Rome and Avorn, 2020).

This placebo-controlled, randomized trial aims to evaluate the therapeutic potential and safety of HCQ in the treatment of adult patients with PCR-confirmed Covid-19 infection in a primary open-care setting. The present trial differs from many other HCQ trials in terms of targeting an earlier stage of the disease, i.e. patients seeking medical care in a primary health care setting because of suspected Covid-19 infection. It is anticipated that HCQ has better efficacy when treatment with the drug is started in the early stage of the disease. In addition, the Finnish public health care system and Finland’s population-based registries provide a unique platform to address this very urgent unmet medical need. Therefore, the study is considered to be warranted and ethically justified.

1.3 Risk benefit assessment

The most common side effects of HCQ include loss of appetite, affect lability, headache, dizziness, tiredness, eye disorders, nausea and abdominal pain. Also serious side effects are possible, such as prolongation of the QT interval, torsades de pointes tachycardia, or retinopathy that might be associated with changes in the retinal pigment epithelium or visual field defects. However, the risk of retinopathy is negligible when the duration of treatment is only seven days. Because of the risk of QT prolongation and torsades de pointes tachycardia, a pre-existing long QT interval and the concomitant use of other drugs that prolong the QT interval will exclude patients from this study (see e.g. Oxiklorin Summary of Product Characteristics and Appendix 2; see also Mehra et al., 2020, and Mercuro et al., 2020).

Covid-19 is a potentially lethal viral disease, and there are no available established specific treatments against it. HCQ may have potential to modify the course of the Covid-19 disease in the early phases of the infection, so it is possible that subjects will derive direct medical benefit from their participation in the study. Reduction of the risk of hospitalization would significantly help to reduce the burden to health care systems caused by the Covid-19 pandemic, and the benefits of such a treatment would have very large societal and economic consequences.

2 STUDY OBJECTIVES AND ENDPOINTS

2.1 Primary objective

The primary objective of this study is to evaluate the therapeutic potential of HCQ, dosed at 300 mg b.i.d. on the first day and then 200 mg b.i.d. for six days (total dose,

3000 mg), in the treatment of adult patients with PCR-confirmed Covid-19 infection in a primary open-care setting, as compared to placebo.

2.2 Secondary objectives

The secondary objectives of this study are:

- to evaluate the safety and tolerability of HCQ, dosed at 300 mg b.i.d. on the first day and then 200 mg b.i.d. for six days (total dose, 3000 mg), in the treatment of adult patients with PCR-confirmed Covid-19 infection in a primary open-care setting, as compared to placebo;
- to collect experience of the use of HCQ in the treatment of Covid-19 infection in outpatients, in order to be able to identify patient characteristics that predict specific treatment responses (favourable or unfavourable); this objective will also be addressed by post-hoc subgroup analysis of the study results and by meta-analysis of pooled patient data from other clinical trials of HCQ in outpatients. The subjects' informed consent covers such further use of the study data; primarily, meta-analysis has been planned with two investigator-initiated open-care trials of HCQ carried out in Germany (see COMIHY, www.ClinicalTrials.gov NCT04340544, and COVID65plus, NCT04351516); and
- to evaluate the impact of Covid-19 infection and its treatment on the mental health and well-being of the study participants.

2.3 Exploratory objectives

In addition, if the data allow, the study has the following exploratory objectives:

- to evaluate the extent and duration of SARS-CoV-2 viral shedding, as evaluated with PCR testing of nasopharyngeal swab samples in study subjects treated with HCQ, as compared to placebo;
- to evaluate the extent and time course of SARS-CoV-2 virus-specific antibody responses in serum of study subjects treated with HCQ, as compared to placebo;
- to evaluate the other possible biomarker changes in blood in study subjects treated with HCQ, as compared to placebo;
- to explore the possible effects of genetic variation in drug metabolizing enzymes on HCQ-related outcomes in the study population;
- to explore the associations of HCQ-related outcome variables with other patient characteristics, e.g. HLA haplotypes, HCQ concentrations, demographic variables, disease history and concomitant medications.

2.4 Primary endpoint

The primary endpoint of the study is:

- number of hospitalizations due to Covid-19 infection within four weeks of entry into the study; the study hypothesis is that treatment with HCQ will reduce the risk of hospitalization because of Covid-19 infection, and the sample size estimate of the study is based on the need to test this hypothesis.

2.5 Secondary endpoints

The secondary endpoints of the study are:

- duration and severity of Covid-19-related symptoms, as reported by the participants in the form of daily self-assessments;
- number of Intensive Care Unit treatment episodes due to Covid-19 infection within four weeks of entry into the study;
- number of deaths due to Covid-19 infection within four weeks of entry into the study;
- number of hospitalizations due to Covid-19 infection within three months of entry into the study;
- number of Intensive Care Unit treatment episodes due to Covid-19 infection within three months of entry into the study;
- number of deaths due to Covid-19 infection within three months of entry into the study;
- number of treatment-related adverse events (AEs) and serious AEs (SAEs);
- number of participants with treatment-related AEs and SAEs;
- all-cause mortality within six months of entry into the study (including post-study follow-up of consenting participants);
- all-cause hospitalizations within six months of entry into the study (including post-study follow-up of consenting participants); and
- self-assessed symptoms of anxiety of the study participants, as assessed with repeated administration of the Generalized Anxiety Disorder 7-item Scale (GAD-7).

2.6 Exploratory endpoints

The exploratory endpoints of the study include the following:

- extent and duration of SARS-CoV-2 viral shedding, as evaluated with PCR testing of nasopharyngeal swab samples;
- extent and time course of SARS-CoV-2 virus-specific antibody responses in serum;
- possible other biomarker changes in blood in study subjects.

3 STUDY DESIGN

3.1 Type and design of the study

This is a phase 2, placebo-controlled, double-blind, randomized, parallel-group treatment trial, performed at several study sites in a primary public health care setting. The study will be started in the region of Satakunta in Finland, but it may be expanded to other regions of Finland. Up to 600 evaluable subjects are planned to participate in the trial. The subjects will be randomized to receive either either HCQ capsules at 300 mg b.i.d. for one day and then 200 mg b.i.d. for 6 days, or placebo capsules for 7 days.

3.2 Randomization and blinding

This is a double-blind study. The participants and study personnel will remain blinded to the treatment allocation by having both investigational medicinal products (IMPs) packed in identical containers. Masking of the treatments is performed by re-

formulation of the IMPs so that the HCQ capsules and the placebo capsules have identical appearance.

At the screening visit, subjects who have signed the informed consent form (ICF) will be given screening numbers beginning from S1001. Randomization will be performed when the investigator has confirmed a subject's eligibility. After randomization, subjects will receive subject numbers starting from 1001 and will be allocated to the treatment assigned to each subject number as indicated by the randomization scheme. Detailed information on randomization will be provided in a separate Randomization Plan/Request document prepared by the DM provider.

In case of a medical emergency, the treatment code may be opened in the electronic data management system of the study (Viedoc™). Unblinding by the investigator is restricted to situations when knowledge of the treatment allocation is considered necessary for the safety and medical treatment of the subject. The reason for unblinding will be documented, along with the date and time of unblinding and an identifier of the person who broke the code. In case of a subject's death or hospitalization, that subject's participation in the study is concluded, and the treatment code of that subject will be opened by the Medical Lead of the study. For hospitalized subjects, this information will communicated to the hospital in charge of the patient's subsequent medical care. De-identified unblinded information on deaths and hospitalizations will also be provided to the SC for ongoing evaluation of the participants' safety.

3.3 General study outline

The total duration of subject participation will be approximately 3 months. The study consists of a screening period, a treatment period and a follow-up period.

At the screening visit, after a consent discussion with the investigator, the subjects will first sign the ICF and then undergo other eligibility evaluations, including collecting of a nasopharyngeal swab sample for SARS-CoV-2 PCR analysis, unless a positive PCR result is already available. Subjects will be enrolled and randomized once the results of the screening assessments are available and the investigator has confirmed the subject's eligibility. A positive test result in SARS-CoV-2 PCR analysis of a nasopharyngeal swab sample taken and analyzed outside of this study may be used for evaluation of virus positivity, if taken within five days of enrolment. Enrolment of subjects without previous PCR-confirmed SARS-CoV-2 virus shedding must have to await a positive SARS-CoV-2 PCR test result of a nasopharyngeal swab sample collected at the screening visit. The participants will be contacted by telephone as soon as the screening test results are available and told whether or not they are eligible for enrolment.

After enrolment and randomization, subjects will proceed to the treatment period. Home delivery of the medication will be arranged by the study sites, and eligible subjects are to start to use their study medication immediately upon its delivery. During the treatment period, the subjects will receive HCQ or placebo capsules twice daily during 7 days, in addition to standard care for Covid-19 infection. Daily telephone contacts by the study sites are included in the standard care protocol; also, they will be used for collection of patient-reported information. On Day 8, all subjects will be contacted by telephone for an interview on symptoms and possible AEs. Approximately 2 weeks and 4 weeks after start of drug intake, the subjects will visit their study site for assessments.

The follow-up period will start at the 4-week visit and will last until 3 months after start of IMP intake. During the follow-up period, information on health care services that the subjects have received in public health care is collected from the Care Register for Health Care, and information on possible deaths is collected from death certificates archived by Statistics Finland. All subjects will be contacted by telephone 3 months after start of IMP intake for an interview on possible AEs and other health outcomes.

After the follow-up period, with the subject's consent, additional post-study follow-up information will be collected from the Care Register for Health Care and from death certificates until 6 months after start of IMP intake.

The study outline is presented in Table 1 below.

Table 1. Study outline

Protocol activities	Screening period	Treatment period	Follow-up period
Informed consent	X		
Demography and substance use	X		
Physical examination	X		
12-lead ECG	X		
Vital signs[1]	X	X	
Laboratory safety assessments[2]	X	X	
SARS-CoV-2 PCR analysis of nasopharyngeal sample[2]	X	X	
SARS-CoV-2 antibody titres in serum sample	X	X	
Other Covid-19-related biomarkers in blood samples[3]	X	X	
DNA sample[4]	X		
Eligibility criteria and decision of entry	X[2]	X[2]	
IMP administration		X[5]	
Patient-reported health outcomes	X	X[6]	X
Medical history and current medical conditions	X		
Adverse events (AEs) and serious adverse events (SAEs)	X		
Prior and concomitant treatments	X		
Evaluation of information from the registers			X

[1]Vital signs to be taken at the screening visit include systolic and diastolic blood pressure, heart rate, body temperature, respiratory rate and SpO_2. Vital signs to be recorded at the 2-week visit include body temperature and SpO_2.
[2]Eligibility criteria will be verified and a decision to enter the subject into the study will be made before treatment allocation and dosing; the screening period is completed with a telephone contact between the investigator and the participant at the time when all relevant screening test results become available.
[3]Includes sampling for HCQ concentration analysis
[4]Optional. The DNA sample for pharmacogenetic (PG) evaluations and HLA haplotyping may be collected at any time during the study.
[5]The first 7 days of the treatment period
[6]Includes body temperature and pulse oximetry results recorded at home by the patient, until the 2-week visit

4 STUDY PATIENTS/SUBJECTS

4.1 Source population

The subjects participating in this study will be adult males and females with PCR-confirmed Covid-19 infection, fulfilling all of the inclusion criteria and none of the exclusion criteria.

4.2 Number of study patients/subjects

Approximately 600 subjects are planned to be included in the study. Actual enrolment numbers will depend on the development of the Covid-19 epidemic in Satakunta and other regions of Finland.

4.3 Inclusion criteria

1. Subjects over 40 years of age, or 18-40 years of age with one or both of the following:
   a. diabetes mellitus (type 1 or type 2)
   b. BMI > 35 kg/m^2.
2. Subjects capable of providing independent informed consent and signing the informed consent form (the subjects' capacity to consent should be determined in accordance with applicable professional standards and will be based on the investigator's judgment).
3. Subjects with symptoms typical of Covid-19 infection, according to criteria specified in the study protocol, with or without known or suspected exposure to the SARS-CoV-2 virus. The onset of symptoms must be within 5 days of enrolment. Study medication may only be started once a positive SARS-CoV-2 PCR test results of a nasopharyngeal swab sample is available; if the first PCR test result is negative, a second sample may be collected and analyzed.
4. Female subjects of child-bearing potential must agree to employ a reliable method of contraception until three months after start of IMP intake.
5. Subject must agree not to share medication with the others.

4.4 Exclusion criteria

1. Subjects with suspected severe or moderately severe pneumonia, presenting with any of the following: respiratory rate > 26 breaths/min; significant respiratory distress; or SpO_2 ≤94 % on room air;
2. Subjects requiring treatment in the hospital, according to the treating physician's judgement;
3. Subjects having any contraindication to treatment with HCQ, such as a long QTc interval (>450 ms in men, >470 ms in women), porphyria, epilepsy, severe renal insufficiency;
4. Screening 12-lead ECG results showing evidence of clinically significant disturbances of cardiac rhythm or impulse conduction. Atrial fibrillation, paroxysmal supraventricular tachycardia or supraventricular extrasystoles are not exclusionary;
5. Cardiac insufficiency of NYHA Class 3-4;
6. History of stroke, within 6 months of screening;
7. Subjects with a history of seizures within one year of screening;
8. Subjects with a history of drowning accident;

9. Any psychiatric diagnosis or symptoms (e.g., hallucinations, major depression, delusions, schizophrenia, bipolar disorder) that, in the opinion of the investigator, could interfere with study procedures or assessments or subject safety; prior history of suicidal behaviour or suicide attempt within five years of screening will also exclude the subject;
10. Disorder related to alcohol or drug abuse, as defined in DSM-5-TR, within one year prior to screening;
11. Evidence of current or history of any significant autoimmune disease that, in the opinion of the investigator, could interfere with evaluation of the study results or constitute a health hazard for the subject;
12. Evidence of an immune system that is compromised; including, but not limited to, a diagnosis of HIV; or the subject has been splenectomised or has received an organ transplant (corneal transplants excluded), or is receiving chronic systemic immunosuppressive medication;
13. Evidence of current clinically significant and possibly unstable pulmonary, gastrointestinal, renal, hepatic, endocrine, haematological or cardiovascular system disease or metabolic disturbance;
14. Diagnosis of cancer (haematological or solid tumour) for which the subject is currently being treated, or for which there is evidence of active disease. Subjects with local prostate cancer or local dermatological tumours, such as basal or squamous cell carcinoma, may be included;
15. Any clinically significant abnormalities in laboratory tests, vital signs, ECG or physical examination findings at screening that in the opinion of the investigator require further investigation or treatment or may interfere with study procedures or safety. These may include, but are not limited to, the following:
    a. estimated glomerular filtration ratio (eGFR) < 30 ml/min/1.73 m^2, based on the CKD-EPI equation;
    b. a plasma total bilirubin value >2 times the upper limit of the reference range;
    c. plasma alanine aminotransferase (ALT) or aspartate aminotransferase (AST) value >3 times the upper limit of the reference range;
16. Current or anticipated use or recent prior use (pre-study time limits specified in Section 5.4 of the protocol) of disallowed concomitant treatment; use of concomitant medications that prolong the QTc interval is not allowed (see Appendix 1);
17. Having received in another clinical trial any other investigational medication (unless it can be documented that the subject received only placebo) within 3 months or 5 half-lives (whichever is longer) before screening;
18. Disease or medication that, in the opinion of the investigator, could seriously impact the assessments of safety, tolerability or efficacy.
19. Pregnancy or breast-feeding.

4.5 Recruitment

Possibly eligible adults who contact the study sites to seek medical care for possible Covid-19 infection will be recruited for the study.

At first, the study is briefly described to the subject candidate in layman's language, and the main inclusion and exclusion criteria are checked. If an exclusion criterion is fulfilled, this is immediately told to the candidate, and the interview is terminated with possible health-related instructions. If a positive SARS-CoV-2 PCR analysis result of a nasopharyngeal swab sample is already available at this stage, a screening visit is booked if the candidate might be suitable for the study. If a positive PCR test result is not available, the person is instructed to undergo the standard Covid-19 testing procedure employed by the study site, if indicated on clinical grounds. The study ICF will be presented to subject candidates by suitable and

available methods, and a consent discussion will be carried out at the start of the screening visit. Upon the subject candidate's wish, the subject may then sign and date the ICF in the investigator's presence.

The management of patients with suspected Covid-19 infection or Covid-19-related concerns will not otherwise be impacted by whether or not the person in question is willing to consider participation in this study.

4.6 Instructions concerning lifestyle and concomitant treatments

Female subjects of childbearing potential will have to agree to use a reliable method of contraception until three months after start of IMP intake, if they practice heterosexual intercourse. Reliable contraception methods include reliable oral, transdermal or injectable hormonal contraceptives, intrauterine devices, hormonal intrauterine system, barrier methods (e.g. male or female condom) or having a male partner who is sterile (e.g. vasectomised).

Fasting is not required for the blood tests to be taken on the screening visit, 2-week visit or 4-week visit.

For instructions and restrictions regarding concomitant treatments, please see Section 5.4.

4.7 Withdrawal of subjects

A subject has the right to withdraw his/her consent without any need to justify this decision. However, efforts should be made to find out the reason of the withdrawal. Possible withdrawal will not have any negative effect on the subject's future medical care.

The investigator may withdraw a subject from the study at any time based on the investigator's clinical judgement. A subject will be withdrawn from further IMP dosing if he or she starts a prohibited medication, develops suspected severe or moderately severe pneumonia, requires treatment in hospital, or other inclusion and exclusion criteria are no longer met. A subject will also be withdrawn from further IMP administration if she/he fails to comply with the trial protocol, or an AE jeopardizing her/his well-being occurs, or if the discontinuation is otherwise in the best interest of the subject.

Subjects who discontinue the study drug prematurely will be encouraged to attend all study visits and complete all study assessments. Still, subjects will retain the right to withdraw their consent for the study, and no study procedures should be carried out against their will.

All information collected from a withdrawn subject, including the reason of withdrawal, will be included in the study file for use as study data and for purposes of verification.

5 TREATMENTS

5.1 Investigational treatment

HCQ capsules (re-formulated from Oxiklorin 100 mg tablets, manufactured by Orion Pharma) will be dispensed to the study participants to allow dosing according to the

following regimen: 300 mg twice daily, to be taken with meals, for one day, and then 200 mg twice daily, to be taken with meals, for 6 days. IMPs will be re-formulated, packed and labeled for dispensing by the hospital pharmacy of Satakunta Central Hospital (SataDiag).

5.2 Reference treatment

Placebo capsules (containing cellulose and magnesium stearate) will be dispensed to the study subjects in identical containers as HCQ, to be taken twice daily with meals, for seven days. The placebo capsules will be formulated, packed and labeled for dispensing by the hospital pharmacy of Satakunta Central Hospital (SataDiag).

5.3 Handling of the study products

Manufacturing, packaging, and labelling of the IMPs will comply with GMP regulations. The IMPs are to be stored at below 25°C, out of reach of children.

5.4 Prior and concomitant treatments

All concomitant treatments administered during the study, including the post-treatment period, must be recorded on the CRF page for concomitant treatments. In order to assess subject eligibility, medications used within 4 weeks prior to the screening visit will be recorded.

No other investigational treatment is allowed to be used during the study.

Use of immunosuppressive drugs, such as methotrexate, oral glucocorticoids and interferon products, is prohibited during the treatment period and four weeks before the start of the study treatment.

Use of concomitant medications that may prolong the QT interval is not allowed during the treatment period and in the two weeks before the start of the study treatment. Medications prohibited because of their possible effects on the QT interval are listed in Appendix 2 of this study protocol; a Finnish-language version of this list is also included as an appendix of the ICF.

5.5 Procedures for monitoring of subject compliance

Treatment compliance will be evaluated by asking the participants to register any deviations from the planned treatment regimen in a participant diary to be collected at the 2-week visit and by performing concentration analysis of HCQ in blood samples collected at the 2- and 4-week visits.

6 VISIT SCHEDULE

6.1 Screening

At the screening visit, the potential study subject will first receive written and verbal information on the study and will have an opportunity to ask questions. If a subject decides to participate in the trial, written informed consent will be obtained before any study-related procedures are performed. A copy of the signed ICF will be provided to the subject.

At screening, subjects will be interviewed for their medical and surgical history, use of medications and current medical conditions, including symptoms of Covid-19 infection and possible exposure to the SARS-CoV-2 virus. The screening interview includes a review of the subject's mental health status, with particular attention to suicidal behaviour and suicide attempts in the past five years. Based on the subject's consent given in the ICF, any existing relevant medical records of the subject will be reviewed and copies thereof will be filed in the subject's source data file. A nasopharyngeal swab sample for SARS-CoV-2 PCR analysis will be taken from those subjects who have not yet undergone such testing for Covid-19 infection; most study participants will already have a positive SARS-CoV-2 PCR analysis result available at this time. Each subject will undergo a physical examination. Blood samples will be collected for screening laboratory tests and for later analysis of SARS-CoV-2 antibodies and other Covid-19-related biomarkers. Demographic data, 12-lead ECG and vital signs will be recorded, and the GAD-7 questionnaire will be completed by the subject. For a more detailed description on the information to be collected at the screening visit, please refer to Section 7.1.

Subjects will be enrolled and randomized once the results of the screening assessments are available and the investigator has confirmed the subject's eligibility. A positive test result in SARS-CoV-2 PCR analysis of a nasopharyngeal swab sample taken and analyzed outside of this study may be used for evaluation of virus positivity, if taken within five days of enrolment. Enrolment of subjects without previous PCR-confirmed SARS-CoV-2 virus shedding must have to await a positive SARS-CoV-2 PCR test result of a nasopharyngeal swab sample collected at the screening visit. If the first PCR test result is negative, a second sample should be collected and analyzed.

As all screening test results will not be available at the screening visit, the participants will be contacted by telephone on the next day (or as soon as the screening test results are available) and told whether or not they are eligible for enrolment. Health-related instructions will be given, and eligible subjects will be told that they should start to use their study medication immediately upon its delivery. Home delivery of the medication will be arranged by the study sites.

6.2 Treatment period

The treatment period will last 4 weeks and the actual treatment 7 days. During the treatment period, the subjects will receive HCQ or placebo capsules twice daily with meals during 7 days. In addition, all subjects will receive standard care for their Covid-19 infection, according to local treatment practice. This includes daily telephone contacts with patients who have symptoms. The script of the daily telephone interviews includes questions on possible suicidal ideation and impairment of mental health. Should the answers to these questions reveal significant adverse changes from baseline or an elevated risk for self-harm, the investigator will be informed, and will then take the necessary actions to provide appropriate psychiatric evaluation and care for the subject, without delay. The subjects will be provided with finger-tip pulse oximetry devices and with instructions on their use during the first 14 days of the treatment period.

On Day 8, all subjects will be contacted by telephone for an interview on symptoms, concomitant medications and possible AEs.

Approximately 2 weeks and 4 weeks after start of drug intake, the subjects will visit their study site for assessments. Safety laboratory tests will be taken at these visits, as well as nasopharyngeal swab samples for SARS-CoV-2 PCR analysis and blood samples for analysis of SARS-CoV-2 antibody titres, other Covid-19-related biomarkers and HCQ concentration analysis. The GAD-7 questionnaire will be completed by the subjects. In addition, they will be asked about symptoms, concomitant medications and possible AEs that might have occurred since the last contact. At the 2-week visit, body temperature and SpO_2 will also be measured, and the participant diaries will be collected.

Because of the slow elimination of HCQ, the treatment period of this study is defined as starting from the first IMP intake and ending at the 4-week visit.

6.3 Follow-up period

The follow-up period will start at the 4-week visit and will last until 3 months after start of IMP intake. During the follow-up period, information on health care services that the subjects have received in public health care is collected from the Care Register for Health Care, and information on possible deaths is collected from death certificates archived by Statistics Finland.

At 3 months after the start of IMP intake, all subjects will be contacted by telephone for an interview on possible AEs and concomitant medications. This telephone contact will conclude the study participation.

After the follow-up period, with the subject's consent, additional post-study follow-up information will be collected from the Care Register for Health Care and from death certificates archived by Statistics Finland until 6 months after start of IMP intake.

7 ASSESSMENTS

7.1 Screening data

7.1.1 Demographic and other baseline information

Information to be recorded during the screening visit includes:

- subject's name, personal identity number, address (only for use at the site; not to be entered into the electronic case report forms (eCRF));
- ethnic origin;
- habits (for the use of nicotine, alcohol, illicit drugs, special diets);
- vital signs (seated or supine blood pressure and heart rate after 10 min rest, tympanic body temperature, respiratory rate, SpO_2);
- 12-lead ECG results;
- results of the physical examination, including body height and weight and chest auscultation findings;
- answers to the GAD-7 questionnaire;
- information on symptoms of Covid-19 infection and possible exposure to the SARS-CoV-2 virus;
- information on previous diseases; the screening interview includes a review of the subject's mental health status, with particular attention to suicidal behaviour and suicide attempts in the past five years;
- information on previous medications; prescription medications 4 weeks before the screening visit will be recorded; over the counter medications, herbal

remedies, trace elements, vitamins, within 4 weeks before the screening visit will be recorded;
- information on previous participation in clinical trials;
- name and information of the subject's next-of-kin (not into eCRF; this information will be removed from the subject's source data after the study).

7.1.2 Screening laboratory safety determinations

Screening laboratory safety assessments include:

- P-Alanine aminotransferase (ALT)
- P-Aspartate aminotransferase (AST)
- P-Bilirubin, total
- P-Creatinine
- Glomerular filtration ratio (eGFR), estimated (based on CKD-EPI equation)
- C-reactive protein (CRP)
- Complete blood count

7.1.3 Covid-19 related laboratory assessments

Covid-19 related laboratory assessments include:

- result(s) of SARS-CoV-2 PCR analysis of nasopharyngeal swab sample(s)
- sample collection for analysis of SARS-CoV-2 antibodies in serum
- sample collection for analysis of other Covid-19-related biomarkers (e.g. P-Ferritin, P-FIDD, P-IL6) in serum or plasma samples.

7.2 Assessment of efficacy

7.2.1 Evaluation of patient records

The patient records will be evaluated, including information collected from health-related registers (the Care Register for Health Care and death certificates archived by Statistics Finland), until 6 months after start of IMP intake to determine the following endpoints:

- number of hospitalizations due to Covid-19 infection within four weeks of entry into the study;
- number of Intensive Care Unit treatment episodes due to Covid-19 infection within four weeks of entry into the study;
- number of deaths due to Covid-19 infection within four weeks of entry into the study;
- number of hospitalizations due to Covid-19 infection within three months of entry into the study;
- number of Intensive Care Unit treatment episodes due to Covid-19 infection within three months of entry into the study;
- number of deaths due to Covid-19 infection within three months of entry into the study;
- all-cause mortality within six months of entry into the study including post-study follow-up of consenting participants);
- all-cause hospitalizations within six months of entry into the study (including post-study follow-up of consenting participants).

7.2.2 Patient-reported health outcomes

During the first two weeks of the treatment period, the subjects are asked to report their symptoms (including body temperature and SpO_2) in the form of daily self-assessments, in order to determine the following endpoint of the study:

- duration and severity of Covid-19-related symptoms.

In addition, the mental health and well-being of the participants will be monitored by asking them to complete the GAD-7 questionnaire at the screening visit and at the 2- and 4-week visits. The script of the daily telephone interviews includes questions on possible suicidal ideation and impairment of mental health. Should the answers to these questions reveal significant adverse changes from baseline or an elevated risk for self-harm, the investigator will be informed, and will then take the necessary actions to provide appropriate psychiatric evaluation and care for the subject, without delay.

7.2.3 SARS-CoV-2 PCR analysis

Nasopharyngeal swab samples are collected for SARS-CoV-2 PCR analysis at the screening visit (unless a previous positive test result is available) and the 2- and 4-week visits, in order to determine the following exploratory endpoint of the study:

- extent and duration of SARS-CoV-2 viral shedding.

7.2.4 Analysis of antibodies and biomarkers

Serum and plasma samples are collected for analysis of SARS-CoV-2 antibodies and other Covid-19-related biomarkers (e.g. P-Ferritin, P-FIDD, P-IL6) at the screening and the 2- and 4-week visits, in order to determine the following exploratory endpoints of the study:

- extent and time course of SARS-CoV-2 virus-specific antibody responses in serum;
- possible other biomarker changes in blood in study subjects.

7.3 Assessment of safety

7.3.1 Clinical safety assessments

During the treatment period, body temperature and SpO_2 will be measured at the 2-week visit.

7.3.2 Laboratory safety assessments

Please refer to Section 7.1.2 for laboratory safety assessments to be performed during the screening visit. The same laboratory assessments will be repeated at the 2-week and 4-week visits, in addition to sample collection for later biomarker and HCQ concentration analysis.

7.3.3 Adverse events

AEs will be collected from informed consent until the end of the follow-up period. During the study visits, the subjects are asked about AEs with non-leading questions. Outside of the visits, daily telephone contact will be maintained with all subjects who have symptoms, and all subjects are instructed to contact the study personnel if they experience a serious or significant AE, if their condition worsens or if they are hospitalized for any reason during the treatment period. In case of minor AEs, the subjects are to report them on their next visit to the study site or during their next telephone contact.

Definitions of adverse events (AE) and serious adverse events (SAE) and the documentation and reporting of them within this study follow GCP, EU and Finnish national guidance.

Adverse Events
An AE is defined as any untoward medical occurrence experienced by a subject, whether or not considered drug related by the investigator. For the current study, all AEs will be coded using MedDRA (version 23.1) by 4Pharma.

AEs are:

- Unfavourable changes in general condition.
- Subjective or objective signs/symptoms.
- Concomitant diseases or accidents.
- Clinically relevant adverse changes in laboratory parameters observed in a subject in the course of a clinical study.

AEs comprise all disturbances of general health status, subjective and objective disease symptoms (including significant, clinically relevant laboratory abnormalities), concomitant diseases, and accidents observed in the context of a clinical trial, irrespective of a possible causal relationship with IMP administration.

All AEs, whether volunteered, elicited, or noted on physical examination, will be recorded throughout the study, i.e., from the screening visit until the end of study participation. AEs that occur prior to the first study product administration will be reported separately from treatment-emergent adverse events, which will be of particular interest.

When an AE occurs, the first decision will be to determine its severity and relationship to the study products.

The severity of AEs will be categorized as follows:

- Mild = Experience is minor and does not cause significant discomfort to subject or change in activities of daily living (ADL) compared to subject baseline; subject is aware of symptoms but symptoms are easily tolerated.
- Moderate = Experience is an inconvenience or concern to the subject and causes interference with ADL compared to subject baseline but the subject is able to continue with ADL.
- Severe = Experience significantly interferes with ADL and the subject is incapacitated and/or unable to continue with ADL compared to subject’s baseline.

The relationship of an AE to study products will be categorized as follows:

- Related; when there is a reasonable possibility of a causal relationship between study product and an AE (i.e. adverse drug reaction, ADR);
- Not related; when an AE does not follow a reasonable temporal sequence from product administration or when an AE can be reasonably explained by other factors including underlying disease, concomitant drugs or concurrent treatment;
- Study treatment not initiated.

The investigator marks on the AE-SD form all AEs, indicating his/her assessment of a causal relationship to study treatments using the categories above.

As far as possible, each AE will also be described by its duration (start and end date and time), and the action(s) taken, and, as relevant, the outcome.

Serious Adverse Events
A Serious Adverse Event (SAE) is any adverse event occurring during the study that results in any of the following outcomes:

- Death.
- A life-threatening AE that placed the subject, in the view of the investigator, at immediate risk of death.
- Inpatient hospitalization or prolongation of existing hospitalization.
- A persistent or significant disability/incapacity.
- A congenital anomaly/birth defect.
- Important medical event that may require medical or surgical intervention to prevent one of the above outcomes.

An important medical event that may not result in death, be life-threatening, or require hospitalization may be considered an SAE when, based upon appropriate medical judgment, it may jeopardize the subject and may require medical or surgical intervention to prevent one of the outcomes listed in this definition.

In case of an SAE, the investigator will contact the Sponsor’s designated Medical Lead as soon as possible. SAEs must be reported within 24 hours of becoming aware of an SAE, regardless of the time that may have elapsed since the time the event occurred and regardless of the causal relationship of IMP to the event. An initial SAE report will be submitted to the Sponsor (and Fimea) as agreed between the parties, and a follow-up report will be provided later, if indicated. Follow-up reports to an SAE should be prepared if any relevant change in the condition of the study subject occurs after the initial report. SAEs should be followed up until resolved or until the event is considered chronic and/or stable outcome.

The contact person and address of the Sponsor for SAEs are:

, MD, Porin perusturvayhtymä
e-mail @fimnet.fi
tel. +358 40

Suspected Unexpected Serious Adverse Reactions
An unexpected adverse drug reaction is any ADR, the specificity or severity of which is not consistent with the summary of product characteristics of HCQ. Suspected unexpected serious ADRs (SUSARs) are subject to expedited reporting to Fimea. The investigator reports all authority-reportable AEs to the Sponsor and to Fimea, as agreed between the parties.

7.4 Assessments of pharmacokinetics, pharmacogenetics and immunogenetics

Blood samples will be collected at the 2- and 4-week visits in order to monitor treatment compliance by concentration analysis of HCQ. The HCQ concentration results will additionally be used to perform population pharmacokinetic modeling in order to evaluate associations between drug exposure and relevant patient outcomes.

A separate whole-blood sample (with EDTA as anticoagulant) will be collected from consenting participants for later DNA extraction and pharmacogenetic (PG) analysis of genetic variation in drug metabolizing enzymes and exploration of immunogenetic associations in the study outcomes. The PG samples will be tested with the Abomics PGx test platform that includes the genes for ALDH2, BCHE, CYP1A2, CYP2B6, CYP2C19, CYP2C9, CYP2D6, CYP3A5, DPYD, F2, F5, G6PD, IFNL3, SLCO1B1, TPMT, UGT1A1 and VKORC1, of which at least CYP2D6 is known to be involved in the metabolism of HCQ. The PG results will be used for interpretation of the HCQ-related outcomes of the study. Immunogenetic analysis is carried out for HLA haplotypes, but it may be expanded to other genes related to immune functions and susceptibility to Covid-19 infection and its manifestations. The blood sample for DNA extraction may be collected at any time during the study. The PG analysis and the immunogenetic analysis are exploratory and optional, and will only be carried out if sufficient numbers of samples become available for meaningful analysis.

8 STATISTICS AND DATA MANAGEMENT

8.1 Estimation of sample size

Up to 600 subjects will be included in the study. The number of hospitalizations due to Covid-19 infection within four weeks of entry into the study is a main outcome measure of the current study. Assuming a hospitalization rate of 15 % in the study population, and a 50 % reduction in the risk for hospitalization in the group treated with HCQ, a treatment group size of 278 provides 80 % power to detect a statistically significant treatment effect at $p < 0.05$. Since the other efficacy-related evaluations are of secondary/explorative nature in this study, no formal sample size calculations have been performed for these endpoints. For a hospitalization risk of 10 %, the group size required for 80 % power would be 435, and for a risk of only 5 %, 906 subjects per group would be required for 80 % power. As the true risk of hospitalization in the study population is unknown, and as an effect size of less than 50 % may also be considered clinically significant, both expansion of the trial beyond the initial catchment region of Satakunta and meta-analysis by combining the study results with those derived from other similar patient populations is intended.

Detailed information on the power calculations will be provided in a separate Statistical Analysis Plan (SAP). The power calculations were made using nQuery Advanced version 8.5.

8.2 Statistical methods

A summary of the statistical methods is given below.

8.2.1 Statistical Analysis Plan

Detailed statistical analysis information will be provided in the SAP before database lock.

8.2.2 Statistical hypotheses

The number of hospitalizations due to Covid-19 infection within four weeks of entry into the study is the primary outcome measure of the study. The following null hypothesis will be tested corresponding to this endpoint:

H_0: there is no difference in the number of hospitalizations due to Covid-19 infection within four weeks of entry into the study between the HCQ and placebo groups.

The null hypothesis will be tested against the following alternative hypothesis:

H_1: treatment with HCQ will reduce the risk of hospitalization because of Covid-19 infection within four weeks of entry into the study.

The evaluations for secondary and exploratory endpoints will mainly be based on descriptive statistics.

8.2.3 Data sets to be analyzed

A subject classification document including a list of protocol deviations with a clinical classification of the deviations and the definition of the analysis datasets will be prepared and approved after database lock before opening of the randomization code.

Three main analysis data sets will be constructed. The intention-to-treat (ITT) data set will include all enrolled subjects. The safety data set will consist of all subjects having received at least one dose of study medication. The per-protocol (PP) dataset including all subjects with SARS-CoV-2 PCR-confirmed Covid-19 infection who received at least four days of treatment with study medication and for whom evaluable outcome data are available.

8.2.4 General statistical considerations

The data will primarily be presented with summary statistics. Summary statistics will include at least number of subjects, mean, standard deviation, median, minimum and maximum for continuous data, and frequency and percentage for categorical data. All data collected will be listed by subject and treatment group.

8.2.5 Demographic and baseline characteristics

The demographic and baseline characteristics of the subjects will be presented using descriptive summary statistics.

8.2.6 Analysis of efficacy variables

For the primary variable of the study, the proportion of hospitalizations will be estimated within each treatment group. The main analysis will be made using a logistic regression model. The methods of analysis of all efficacy variables will be

provided in the SAP. At least, analysis of all primary and secondary efficacy variables will be performed for both the PP and ITT datasets.

8.2.7 Analysis of safety variables

8.2.7.1 Evaluation of adverse events

AEs will be collected starting from the moment of the ICF signature, but AEs occurring before and after IMP administration will be reported separately. All AEs reported during the study will be classified by system organ class and preferred terms using the Medical Dictionary for Regulatory Activities (MedDRA) coding system, and according to severity of the AE (mild, moderate, severe) and causality to the IMP. The number and proportion (%) of subjects having each AE and the number of events will be tabulated overall and by treatment group.

8.2.7.2 Clinical safety evaluations

The clinical safety data will primarily be analyzed using summary statistics. Physical examination findings will be evaluated with descriptive statistics. The actual values of systolic and diastolic blood pressure, heart rate, body temperature, SaO_2 and respiratory rate will be presented using summary statistics. 12-lead ECG results will be summarized and tabulated as normal, abnormal, not clinically significant and abnormal, clinically significant.

8.2.7.3 Clinical safety laboratory evaluations

Laboratory safety variables and their changes from baseline will be summarized using descriptive statistics and categorized as normal, abnormal, not clinically significant and abnormal, clinically significant. The values will also be categorized into low, normal and high according to their reference ranges. Clinically significant laboratory abnormalities will be reported in the study report.

8.2.8 Analysis of pharmacokinetic, pharmacogenetic and immunogenetic variables

The pharmacokinetic, pharmacogenetic and immunogenetic study results will not be entered into the eCRF, and these results will be analyzed and reported separately by the participating analysis service providers and collaborating laboratories. These reports will be included as appendices into the main clinical study report.

The pharmacokinetic analysis is performed to confirm drug exposure and to assess C_{max} and rate of drug elimination. No thorough pharmacokinetic inferences will be aimed at.

8.3 Data management

Detailed information on data management (DM) will be given in a separate Data Management Plan specifically written for this study.

8.3.1 Database design

The study database and data entry screen design, as well as edit checks will be defined according to the corresponding eCRF and the study protocol.

8.3.2 Data entry

Data will be collected on source data forms and entered into an eCRF designed in accordance with the study protocol. The investigator or designated study site personnel will enter subject data into the eCRF as soon as possible during or after the subject’s visit. All data entries in the eCRF should be consistent with the source documents. The investigator will electronically sign the data to confirm that the information is accurate and correct.

8.3.3 The query process

The Study Monitor will review the eCRFs and compare them with the relevant source documents to ensure the validity and accuracy of the study data. If corrections are needed, the Study Monitor will raise a query in the eCRF. The investigator or designated member of the study site personnel will answer any queries and make the needed corrections or alterations.

8.3.4 Medical encoding

AEs and medical history verbatim terms will be coded using MedDRA (version 23.1).

8.3.5 Database lock

When all data have been entered and discrepancies solved, the study database can be locked. The locked database is used in the final statistical analyses and in the reporting of the study results.

8.4 Software

Viedoc™ (Viedoc Technologies AB, Uppsala, Sweden) will be used as an electronic data capture (EDC) system for this study.

9 QUALITY CONTROL AND QUALITY ASSURANCE

During the clinical execution of the trial, the principles of GCP are followed throughout the study. For data management and statistics, the standard operating procedures of 4Pharma are followed.

The analyses of safety laboratory assessments will be performed at the laboratory of SataDiag. The analysis of HCQ concentrations in whole blood samples is planned to be performed at the Bioanalytical Laboratory of the University of Turku, Institute of Biomedicine. Diagnostic PCR testing will be carried out at SataDiag and antibody testing from serum will be performed by collaborating researchers at TYKSLAB and the University of Turku. Optional pharmacogenetic testing for drug metabolizing enzymes will be carried out by Abomics Ltd. HLA haplotyping will be performed by TYKSLAB, the accredited hospital laboratory of Turku University Hospital. Laboratory quality certificates will be available.

The study will be monitored by Turku Clinical Research Centre (TurkuCRC). The monitor is allowed to monitor the study as frequently as necessary to ensure that the data recording and protocol adherence are satisfactory. The eCRFs and related source data will be reviewed in sufficient detail.

A curriculum vitae in English will be obtained from all investigators who sign the protocol and from other relevant persons.

10 ETHICAL CONSIDERATIONS

This study will follow the relevant regulations and guidance for biomedical research involving human subjects, such as the Declaration of Helsinki, GCP, and national and EU legislation. Special emphasis will be put on the well-being of the subjects.

Prior to initiation of the study, the study protocol, the subject information leaflet and the ICF and the texts of any advertisements used for the recruitment of study subjects will be submitted to and approved by an independent Ethics Committee (EC). Additionally, the EC will be notified of any other possible materials to be given to the subjects (e.g. study subject diary, participant card). The study will be authorized by the CA (the Finnish Medicines Agency, Fimea) before its commencement.

The study subject candidates will be provided with both verbal and written information on the study, its risks and benefits. They are encouraged to ask questions on the study. After having had enough time to consider their participation they may sign the ICF. No study procedure will be implemented prior to obtaining written IC that is signed by the subject at the time of consent. A copy of the signed ICF will be given to the subject. The investigator will keep each subject’s signed ICF on file for inspection by a regulatory authority at any time.

The subjects are told what information will be collected of them and how. The collection of personal information is described in compliance with the EU General Data Protection Regulation; the description is kept in the ISF and is made available to the subjects, if they wish. The investigator assures that the privacy of the subjects, including their personal identity and all other medical information, will be maintained at all times.

The subjects are urged to report all AEs to the study personnel and they are given phone numbers of the investigator and study nurses whom they are instructed to call if they observe AEs, or in other urgent study-related issues.

A Steering Committee (SC) will provide oversight of the study. One of the tasks of the SC will be to provide recommendations to the Sponsor and the Coordinating Investigator on whether enrolment of participants into the study should be continued or terminated, or whether modifications should be introduced into the study protocol in the form of a substantial amendment. No formal interim analysis of the accumulating study results has been planned, but the SC and the data management (DM) provider of the trial will perform unblinded follow-up of all hospitalizations and all deaths occurring in the study population, in order to be able to call for termination or modification of the trial.

Substantial changes to the final approved study protocol will only be initiated with the EC’s favourable opinion of a written amendment, except when necessary to eliminate immediate hazards to the subjects. When the change involves only logistics or administration, it is considered non-substantial, and needs not be submitted to the EC or the CA. The CA will also be notified before adopting a substantial amendment.

After the study has been completed or terminated, and the study report prepared, the EC and CA will be notified of the study results.

The study procedures are minimally invasive, but study participation involves certain risks for the subjects. The volume of blood to be collected for the purposes of the study is less than 100 ml. Half of the participants will be exposed to treatment with HCQ, a drug known to have adverse effects in some individuals. HCQ-related risks will be minimized by careful subject selection and close follow-up of the participants. The subjects may also derive benefit from their participation if HCQ has efficacy in the treatment of Covid-19 infection. For study site personnel, execution of the study procedures adds to their contacts with patients with Covid-19 infection, and may increase their risk of contracting the disease. Appropriate precautions will be taken at all study sites to minimize this risk.

11 DATA HANDLING AND RECORD KEEPING

11.1 Case report forms

Generated subject data will be recorded on eCRFs provided for this study by the DM provider, 4Pharma Ltd. Only authorized persons are allowed to make eCRF entries. They may also make corrective entries on the eCRFs if the investigator has not yet signed the subject's eCRF. After signing, only the investigator is allowed to make corrections.

An eCRF is required for each study subject. eCRFs will be completed in English by the investigator or other authorized study personnel. The investigator has to confirm the contents of the eCRF by an electronic signature. An audit trail within the system will track all changes/corrections made. Instructions and training for completing the eCRFs will be provided. These instructions will cover the contents and technical issues of the electronic data capture system.

11.2 Source data

Study-specific source data forms will be prepared before study initiation. Access to the source data revealing the identity of the study subjects is only to study site personnel and to the study monitor. The generated source data are stored within each study site's ISF.

The results of the laboratory blood and urine safety determinations are stored as print-outs within the ISF and in the patient records of the study sites and the laboratory of SataDiag, the personnel of which is bound to professional secrecy. The 12-lead ECG print-outs are stored within the site's ISF.

The contact information of the subject's next-of-kin will be destroyed before the site's ISF is archived.

11.3 Deviations

In case the study monitor, an investigator, a study nurse or other authorized person involved in the study observes a protocol deviation or discrepancy, he/she should describe the issue as clearly as possible in a written memorandum. In addition to the date and signature of the author, the investigator, and possibly the monitor and/or authorized Sponsor's representative will also sign the memorandum.

Deviations concerning a single subject will be described on the corresponding eCRF.

11.4 Amendments

Minor changes (e.g., concerning logistics or administration) to the clinical study protocol can be clarified in a memorandum or in a non-substantial amendment, if the change has no effect on the safety of the subjects or on the scientific value of the study. The investigator will inform the Sponsor of such minor changes. All essential changes to the clinical study protocol are described in a substantial amendment, which is submitted for approval by the EC and CA before adopting the changes. Amendments to the clinical study protocol are prepared as agreed by the parties involved in the study.

12 STUDY SCHEDULE

This clinical study is planned to be performed during Q3/2020-Q2/2021, with register-based follow-up of the last enrolled participant until the end of the year 2021.

13 CRITERIA FOR PREMATURE STUDY TERMINATION

The study may be discontinued at the discretion of the Principal Investigator or the Sponsor based e.g. on the occurrence of the following:

- ADRs unknown to date in respect to their nature, severity, and duration or the unexpected incidence of known ADRs;
- New information becomes available that mandates study discontinuation, e.g. for futility or for proof of efficacy of HCQ or another type of treatment of Covid-19 infection in the patient population of this study.
- Other medical or ethical reasons affecting the continued performance of the study.
- Difficulties in the recruitment of subjects.
- Significant deviations from the study protocol.

The investigator will inform the EC and the Sponsor will inform the CA if the study is terminated prematurely. The Sponsor reserves the right to prematurely terminate the study for valid scientific or administrative reasons. The investigator will proceed to appropriate actions concerning the study subjects in the case of premature termination of the study.

A study site may withdraw from further subject recruitment at any time if sufficient resources can no longer be allocated to proper execution of the study procedures. Already enrolled subjects should be treated and followed up according to the study protocol even if a site is withdrawn from further enrollment activities.

Individual study subjects are free to withdraw from further study treatment and from further study procedures if they wish. In addition, subjects should be withdrawn by the investigator from further study medication in case the patient must be hospitalized for any reason, if the subject starts a prohibited medication or if an AE severe enough to justify discontinuation of treatment with HCQ develops. The investigator may withdraw a subject from the study at any time based on the investigator’s clinical judgement. A subject will also be withdrawn from further IMP administration if she/he fails to comply with the trial protocol, or if the discontinuation is otherwise in the best interest of the subject.

Subjects who discontinue the study medication prematurely will be encouraged to attend all study visits and complete all study assessments.

14 FINANCING AND INSURANCE

Financial matters are covered by agreements between the Sponsor and the other relevant parties of the study. The Sponsor follows an insurance policy covering damages caused by the investigational products administered during the study. In case of any injury caused by an incident that is related to the study procedures but is not causally related to the investigational product itself, study subjects will be covered by the patient insurance of the participating study sites.

15 STUDY REPORT AND PUBLISHING

A clinical study report will be prepared after the study has been completed or prematurely terminated. The EC and CA will be notified about study completion or termination according to applicable regulation. The study report will be approved by the PI and the Sponsor. The Sponsor remains the exclusive owner of the study data defined in the protocol. The study results will be submitted for open-access publication according to standard scientific practice and ICJME guidelines. A publication plan will be prepared for review and acceptance by the involved investigators and other parties. Publications will not be submitted for publication without prior approval of the Sponsor.

16 ARCHIVING

The site ISFs (including e.g. source data, subject screening and identification logs, original signed Informed consents, and drug accountability records) will be archived to enable possible follow-up assessments or inspections by regulatory authorities. The ISF is archived for at least 15 years after the end of the study.

Information collected during the course of this study will be stored by the Sponsor and used in the evaluation of HCQ as a treatment for Covid-19, and thereafter for as long as the information is relevant to patient care. Its use includes the transfer of data to regulatory authorities of the European Union or its member states, the USA or other countries for scientific purposes. All information is handled confidentially and according to the current laws and regulations.

The Sponsor will archive the Trial master file according to the current laws and regulations.

REFERENCES

Beigel JH, Tomashek KM, Dodd LE, et al. Remdesivir for the treatment of Covid-19 - Preliminary report [published online ahead of print, 2020 May 22]. N Engl J Med. 2020;10.1056/NEJMoa2007764.

Chen JLL, Liu P, Xu Q, Xia L, Ling Y, Huang D, Song S, Zhang D, Qian Z, Li T,Shen Y, Lu H L.A pilot study of hydroxychloroquine in treatment of patients with common coronavirus disease-19 (COVID-19). J Zhejiang Univ (Med Sci) 2020; 49 0-0.

Chen Z, Hu J, Zhang Z, Jiang S, Han S, Yan D, et al. Efficacy of hydroxychloroquine in patients with COVID-19: results of a randomized clinical trial. medRxiv 2020 Jan 1;2020.03.22.20040758.

COVID-19 Treatment Guidelines. National Institutes of Health; May 12, 2020. Accessed May 30, 2020. https://covid19treatmentguidelines.nih.gov/introduction/

Deshmukh, V.; Tripathi, S.C.; Pandey, A.; Deshmukh, V.; Patil, A.; Sontakke, B.; Vykoukal, J. COVID-19: A conundrum to decipher. Preprints 2020, 2020040019. doi: 10.20944/preprints202004.0019.v2.

Dolin R, Hirsch MS. Remdesivir – an important first step. N Engl J Med. May 27, 2020, DOI: 10.1056/NEJMe2018715

Gautret P, Lagier JC, Parola P, Hoang VT, Meddeb L, Mailhe M, Doudier B, Courjon J, Giordanengo V, Vieira VE, Dupont HT, Honoré S, Colson P, Chabrière E, La Scola B, Rolain JM, Brouqui P, Raoult D. Hydroxychloroquine and azithromycin as a treatment of COVID-19: results of an open-label non-randomized clinical trial. Int J Antimicrob Agents. 2020:105949

Li LQ, Huang T, Wang YQ, et al. Novel coronavirus patients’ clinical characteristics, discharge rate and fatality rate of meta-analysis [published online ahead of print, 2020 Mar 12]. J Med Virol 2020; doi: http://dx.doi.org/10.1002/ jmv.25757.

Liu J, Cao R, Xu M, et al. Hydroxychloroquine, a less toxic derivative of chloroquine, is effective in inhibiting SARS-CoV-2 infection in vitro. Cell Discov 2020; 6: 16.

Lu H, Stratton CW, Tang YW. Outbreak of pneumonia of unknown etiology in Wuhan China: the mystery and the miracle. J Med Virol. 2020; 92. doi: 10.1002/jmv.25678.

Magagnoli J, Narendran S, Pereira F, Cummings T, Hardin JW, Sutton SS, Ambati J. Outcomes of hydroxychloroquine usage in United States veterans hospitalized with Covid-19. medRxiv 2020, doi: 10.1101/2020.04.16.20065920.

Mehra MR, Desai SS, Ruschitzka F, Patel AN. Hydroxychloroquine or chloroquine with or without a macrolide for treatment of COVID-19: A multinational registry analysis. Lancet 2020. doi: 10.1016/S0140-6736(20)31180-6

Mercuro NJ, Yen CF, Shim DJ, et al. Risk of QT interval prolongation associated with use of hydroxychloroquine with or without concomitant azithromycin among hospitalized patients testing positive for Coronavirus Disease 2019 (COVID-19). JAMA Cardiol. Published online May 01, 2020. doi:10.1001/jamacardio.2020.1834

Rome BN, Avorn J. Drug evaluation during the Covid-19 pandemic. N Engl J Med 2020. doi: 10.1056/NEJMp2009457

Rosenberg ES, Dufort EM, Udo T, et al. Association of treatment with hydroxychloroquine or azithromycin with in-hospital mortality in patients with COVID-19 in New York State. JAMA. Published online May 11, 2020. doi:10.1001/jama.2020.8630

APPENDICES

Appendix 1. Signed consent of the investigator
Appendix 2. Medications prohibited because of their possible effects on the QT interval
Appendix 3. Telephone assessment form

Appendix 1: Signed consent of the investigator

**Sponsor:**	Porin kaupunki
**Study code:**	LIBERTY
**Study title:**	Controlled clinical trial of hydroxychloroquine in the treatment of adult patients with Covid-19 infection in a primary care setting
**Study centre:**	
**Name of the investigator:**	

I have read the protocol *(version 2.0/14Jul2020)* and agree to its terms.

Date and location: ____

Signature: ____

Appendix 2: Medications prohibited because of their possible effects on the QT interval

Amiodarone
Anagrelide
Arsenic trioxide
Azithromycin
Chloroquine
Chlorpromazine
Chlorprothixene
Ciprofloxacin
Cisapride
Citalopram
Clarithromycin
Cocaine
Disopyramide
Domperidone
Donepezil
Dronedarone
Droperidol
Erythromycin
Escitalopram
Flecainide
Fluconazole
Haloperidol
Ibutilide
Levofloxacin
Levomepromazine
Levomethadyl acetate
Methadone
Moxifloxacin
Ondansetron
Oxaliplatin
Papaverine HCl (Intra-coronary)
Pimozide
Procainamide
Propofol
Quinidine
Roxithromycin
Sevoflurane
Sotalol
Sulpiride
Terlipressin
Thioridazine
Vandetanib

Source: www.crediblemeds.org

Appendix 3: Telephone assessment form

1) **Yleisvointi asteikolla 0-10 (0 = huonoin mahdollinen; 10 = täysin terve verrattuna nykyistä sairautta edeltävään tilanteeseen)**

2) **Hengenahdistuksen tunne asteikolla 0-10 (0 = ei hengenahdistusta; 10 = paha hengenahdistus levossakin)**

3) **Pulssioksimetrin lukema soiton aikana mitattuna _______ ja aamulla pian heräämisen jälkeen ___________**

4) **Kuumeen mittaustulos yhden desimaalin tarkkuudella aamulla heräämisen jälkeen ______**

5) **Onko muilla samassa taloudessa asuvilla saman sairauden oireita? Kenellä? Milloin ne alkoivat?**

6) **Oletteko tuntenut itsenne sekavaksi, masentuneeksi tai poikkeuksellisen jännittyneeksi viime päivien aikana?**

7) **Lääkkeen mahdolliset haittavaikutukset edellisen kyselyn jälkeen: ____**

8) **AE ja SAE raportointi tutkimuksesta vastaavalle lääkärille (luettelo mahdollisista haittatapahtumista alkamisaikoineen)**

9) **Muut mahdolliset oireet**

Tutkittavan numero tutkimuksessa: _______________________

Puhelinhaastattelun päiväys ja kellonaika. Vastaukset koskevat tätä päivää:

____
pp kk vuosi klo hh.min

Haastattelija (nimikirjoitus ja nimen selvennys):

Tutkimuskeskus(tutkimukseen osallistuva terveyskeskus):
